# Factors influencing oral microbiome analysis: from saliva sampling methods to next-generation sequencing platforms

**DOI:** 10.1038/s41598-023-37246-2

**Published:** 2023-06-21

**Authors:** Eunsik Bang, Sujin Oh, Uijin Ju, Ho Eun Chang, Jin-Sil Hong, Hyeong-Jin Baek, Keun-Suh Kim, Hyo-Jung Lee, Kyoung Un Park

**Affiliations:** 1 Seoul National University College of Medicine, Seoul, Republic of Korea; 2 Department of Laboratory Medicine, Seoul National University College of Medicine, Seoul, Republic of Korea; 3 PHiCS Institute, Seoul, Republic of Korea; 4 Department of Periodontology, Section of Dentistry, Seoul National University Bundang Hospital, Seongnam, Republic of Korea; 5 Department of Laboratory Medicine, Seoul National University Bundang Hospital, Seongnam, Republic of Korea

**Keywords:** Laboratory techniques and procedures, Oral microbiology

## Abstract

The exploration of oral microbiome has been increasing due to its relatedness with various systemic diseases, but standardization of saliva sampling for microbiome analysis has not been established, contributing to the lack of data comparability. Here, we evaluated the factors that influence the microbiome data. Saliva samples were collected by the two collection methods (passive drooling and mouthwash) using three saliva-preservation methods (OMNIgene, DNA/RNA shield, and simple collection). A total of 18 samples were sequenced by both Illumina short-read and Nanopore long-read next-generation sequencing (NGS). The component of the oral microbiome in each sample was compared with alpha and beta diversity and the taxonomic abundances, to find out the effects of factors on oral microbiome data. The alpha diversity indices of the mouthwash sample were significantly higher than that of the drooling group with both short-read and long-read NGS, while no significant differences in microbial diversities were found between the three saliva-preservation methods. Our study shows mouthwash and simple collection are not inferior to other sample collection and saliva-preservation methods, respectively. This result is promising since the convenience and cost-effectiveness of mouthwash and simple collection can simplify the saliva sample preparation, which would greatly help clinical operators and lab workers.

## Introduction

Microbiomes are collections of bacteria, viruses, and fungi that reside within the human body and on skin, and play major roles in normal human physiology and disease^1^. The gut microbiome was one of the first to be explored, and various researches have revealed the relationship between the gut microbiome and diseases. For example, changes in gut microbiotic function and composition play major roles in irritable bowel syndrome, colorectal cancer, and obesity^2^.

The oral microbiome biomass is second only to that of the gut^3^. As for the gut microbiome, changes in oral microbiome function or composition are associated with certain systemic diseases. Oral *Porphyromonas gingivalis* and *Fusobacterium nucleatum* modulate the chronic inflammation associated with, and cellular migration and invasion of, oral squamous cell carcinoma^4^. Oral microbiome richness and diversity were reduced in patients with Alzheimer’s disease^5^. Given the similarity between the oral and gut microbiota, and their relationships with systemic disease, it has been suggested that the oral microbiome could predict gastrointestinal cancer^6^.

Next-generation sequencing (NGS) has facilitated such microbiome explorations via the analysis of DNA in the microbiome group^2^. Despite the effectiveness of NGS on oral microbiome analysis, microbiome data are prone to spurious heterogeneity and unwanted variation^7^. Therefore, an important consideration is the factors that affect the variations in sample measurements, pre-analytical and analytical variability^8^. Especially, gathering high-quality samples for accurate sequencing and analysis has become essential. Being consistent when handling samples is key to minimizing technical variations^9^.

Saliva has been widely used to assess oral microbiome status. Saliva sampling is highly convenient, repeatable, and non-invasive, but standardization of methods especially for microbiome analysis is lacking. There are variations in sampling methods (drooling vs. mouthwash), saliva-preservation methods, and sequencing platforms (long- vs. short-read), which make the standardization of saliva collection protocols difficult. Several studies sought to standardize saliva collection. Fan et al.^10^ compared the oral microbiomes of mouthwash and drooling group, and found no significant difference. Lim et al.^11^ found that different collecting, processing, and DNA preparation methods did not significantly affect the salivary microbiome profile, but the genomic DNA purity differed depending on the extraction kits used. Armstrong et al.^3^ reported that preservatives increased the consistency of oral microbiome data. However, no previous literature has evaluated the effects of the sequencing platforms as well as the saliva collection and preservation methods at the same time. Here, we used different sample collecting methods, saliva-preservation methods, and sequencing platforms to optimize oral sampling and analysis. All samples were subjected to 16s rRNA amplicon sequencing followed by bioinformatic analysis.

## Results

### Effects of collection methods on oral microbiome data

#### Short-read next-generation sequencing (SR-NGS) group

**Figure 1:**
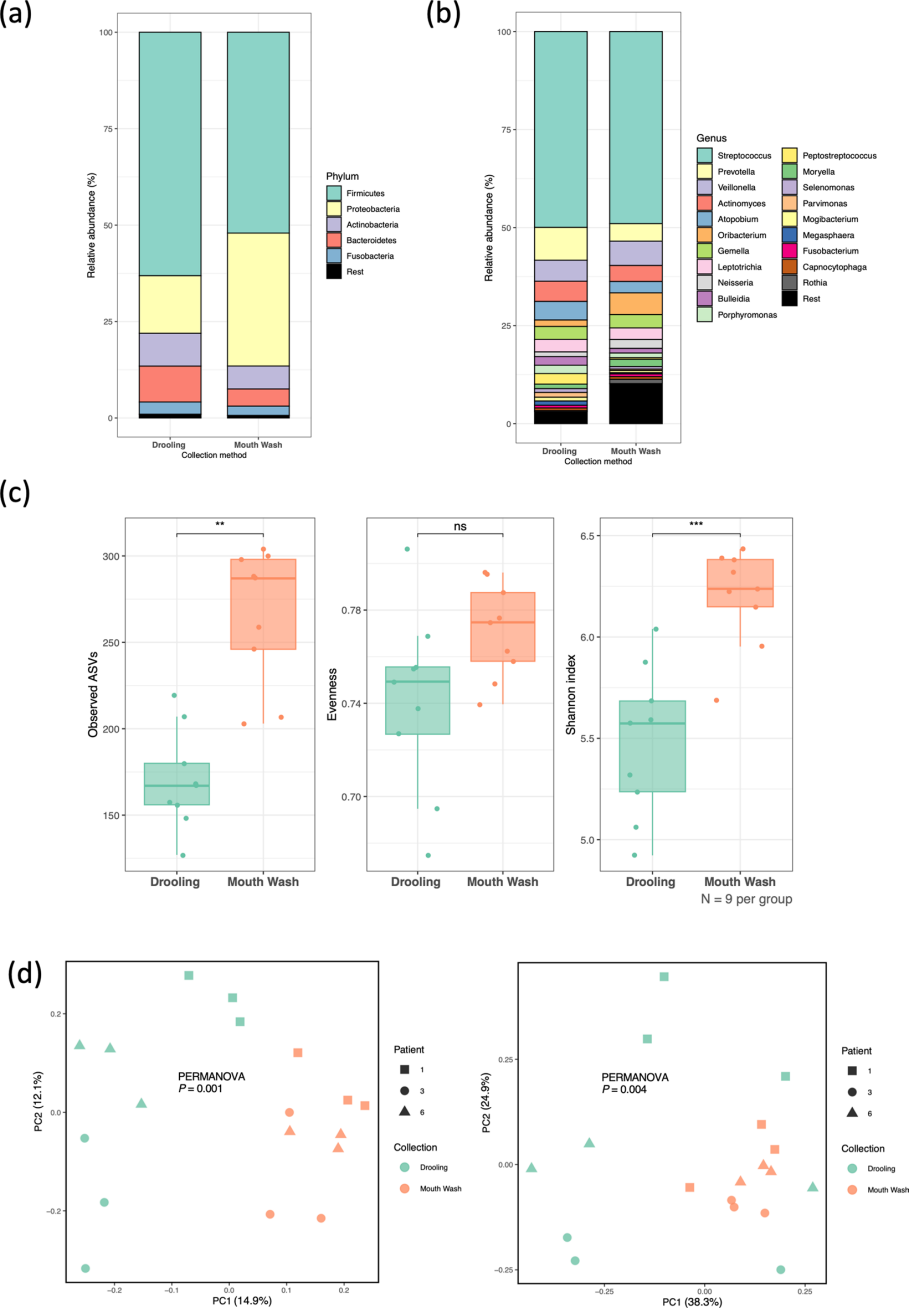
Comparison of data from the drooling and mouthwash groups sequenced by short-read next-generation sequencing (SR-NGS). (**a, b**) Taxonomic barplots showing the taxa and their relative abundances. (**a**) Phylum level, (**b**) Genus level. The five most abundant phylum and 20 most abundant genera were shown based on the mean relative abundance of the two groups. (**c**) Box-and-whisker plots of the alpha diversity indices (observed features, Pielou’s evenness, and Shannon’s entropy). *ns* not significant; **: 0.001 ≤ *P* < 0.01; ***: *P* < 0.001 (**d**) Beta diversities (left: Jaccard distances, right: Bray–Curtis distances) as revealed by principal coordinate analysis (PCoA) plots.

We compared taxonomic composition between drooling and mouthwash samples. The microbial compositions (mean relative abundances at the phylum and genus levels) are shown in Fig. 1a, b. At the phylum level, Firmicutes and Proteobacteria were the first and second most abundant taxa in both groups. At the genus level, *Streptococcus* was the most abundant taxon in both groups (50% and 48% of all sequences, respectively). *Atopobium*, *Bulleidia, Peptostreptococcus,* and *Porphyromonas* showed higher abundances in drooling, while *Neisseria* and *Oribacterium* did in mouthwash samples (Fig. 1a, b, and S1).

In terms of alpha diversity, the number of observed amplicon sequence divergences (ASVs) and Shannon’s entropy were significantly higher in the mouthwash than drooling group (*P* < 0.05), but the community evenness was similar (Fig. 1c). We drew principal component analysis (PCoA) plots of the beta-diversity matrices to explore dissimilarities in the microbial communities between the two collecting methods. The PCoAs of both the Jaccard and Bray–Curtis distances clustered all samples into well-separated drooling and mouthwash groups (permutation multivariate analysis of variance [PERMANOVA]: *R*^2^ = 0.13, *P* = 0.001; and *R*^2^ = 0.17,* P* = 0.004, respectively) (Fig. 1d). Since the two groups exhibited clear dissimilarities of microbial composition, we identified taxa that showed a significant difference in abundance between drooling and mouthwash samples. Three phyla, including Proteobacteria, Cyanobacteria, and GN02 showed differential abundance between the two groups, whereas 19 genera did (adjusted *P* < 0.05) (Table 1).

**Table 1:** Differentially abundant taxa between the drooling and mouthwash samples confirmed by ANCOMBC analysis.

Level	Taxon	W	*p* value	Adj *p* value
Phylum	Cyanobacteria	5.167	< 0.001	< 0.001
	GN02	3.435	0.001	0.003
	Proteobacteria	4.573	< 0.001	< 0.001
Genus	*Acetobacter*	4.631	< 0.001	< 0.001
	*Acinetobacter*	3.558	< 0.001	0.040
	*Actinobacillus*	4.112	< 0.001	0.004
	*Bordetella*	5.149	< 0.001	< 0.001
	*Caulobacter*	19.667	< 0.001	< 0.001
	*Corynebacterium*	5.114	< 0.001	< 0.001
	*Cupriavidus*	13.701	< 0.001	< 0.001
	*Haemophilus*	6.222	< 0.001	< 0.001
	*Lactobacillus*	9.672	< 0.001	< 0.001
	*Listeria*	3.668	< 0.001	0.027
	*Methylobacterium*	4.799	< 0.001	< 0.001
	*Methylotenera*	10.828	< 0.001	< 0.001
	*Microbacterium*	3.805	< 0.001	0.016
	*Olsenella*	4.471	< 0.001	0.001
	*Oribacterium*	4.033	< 0.001	0.006
	*Pseudomonas*	5.920	< 0.001	< 0.001
	*Rothia*	3.567	< 0.001	0.039
	*Staphylococcus*	4.915	< 0.001	< 0.001
	*Xanthomonas*	4.251	< 0.001	0.002

Statistical significance was determined as an adjusted *p* value < 0.05.

*W* generalized log-scaled fold change, *Adj p-value* adjusted *p* value.

#### Long-read next-generation sequencing (LR-NGS) group

**Figure 2:**
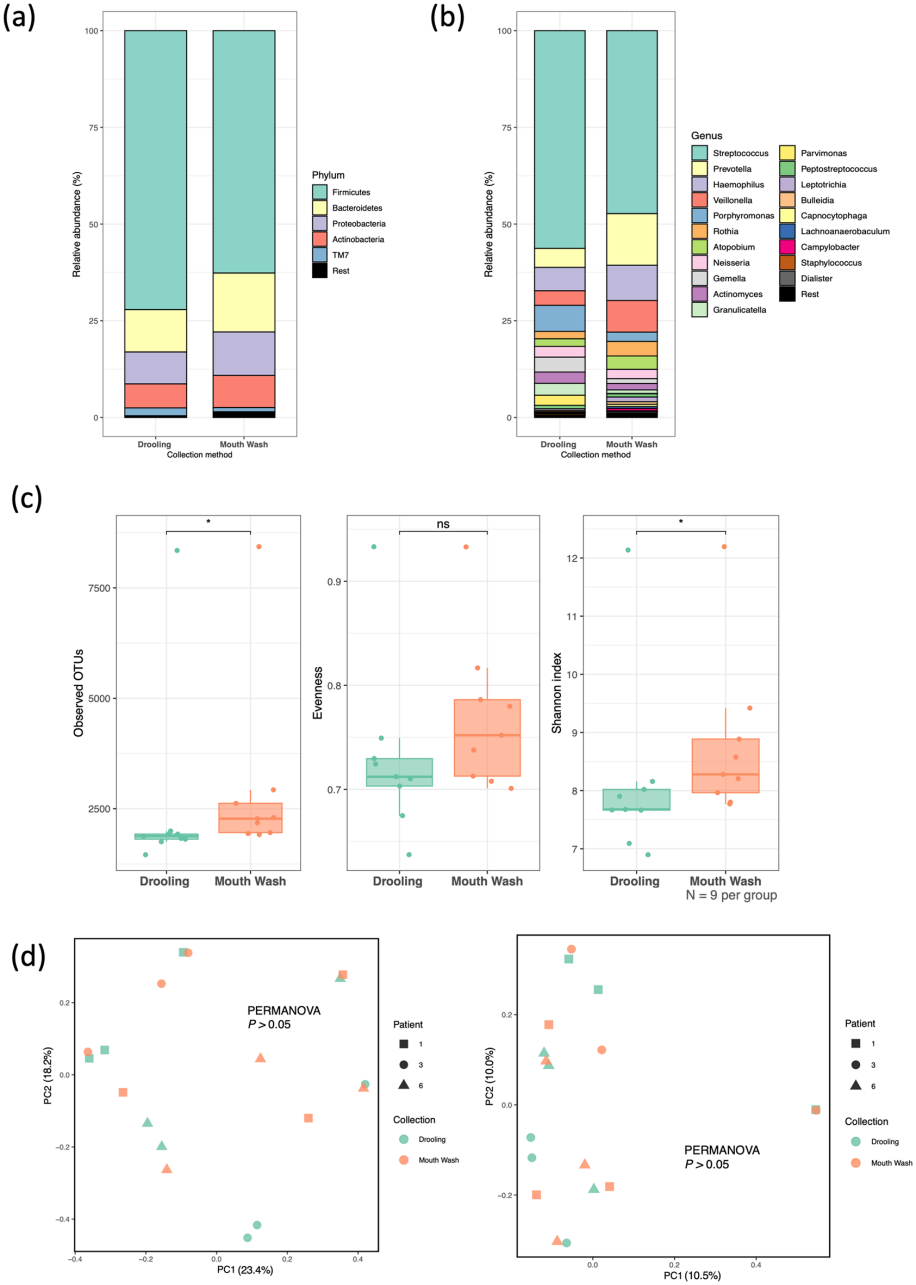
Comparison of the drooling and mouthwash groups sequenced by long-read next-generation sequencing (LR-NGS). (**a, b**) Taxonomic barplots showing the taxa and their relative abundances. (**a**) Phylum level, (**b**) Genus level. The five most abundant phylum and 20 most abundant genera were shown based on the mean relative abundance of the two groups. (**c**) Box-and-whisker plots of the alpha diversity indices (observed features, Pielou’s evenness, and Shannon’s entropy). *ns* not significant; *: 0.01 ≤ *P* < 0.05 (**d**) Beta diversities (left: Jaccard distances, right: Bray–Curtis distances) as revealed by principal coordinate analysis (PCoA) plots.

Taxonomic barplots of the drooling and mouthwash groups are shown in Fig. 2a, b; these barplots illustrate the relative taxon abundances at the phylum and genus levels. In contrast to the data gathered by SR-NGS, Firmicutes and Bacteroidetes were the first and second most common taxa at the phylum level in both groups. At the genus level, *Streptococcus* was the most abundant taxon. *Gemella*, *Porphyromonas*, and *Parvimonas* showed higher abundances in the drooling group, and *Prevotella*, *Veillonella*, *Haemophilus*, *Leptotrichia*, and *Atopobium* did in the mouthwash group.

In terms of alpha diversity, the number of operational taxonomic units (OTUs) was significantly higher in the mouthwash than the drooling group (Wilcoxon rank sum test. *P* < 0.05), as was the Shannon’s entropy (*P* < 0.05). However, similar to the SR-NGS data, the Pielou’s evenness did not differ between the two collection methods (Fig. 2c). In contrast to the SR-NGS data, no significant difference was observed in beta diversity indices (Jaccard and Bray–Curtis distances) between the drooling and mouthwash groups (Fig. 2d).

### Effects of saliva-preservation methods on oral microbiome data

#### SR-NGS group

**Figure 3:**
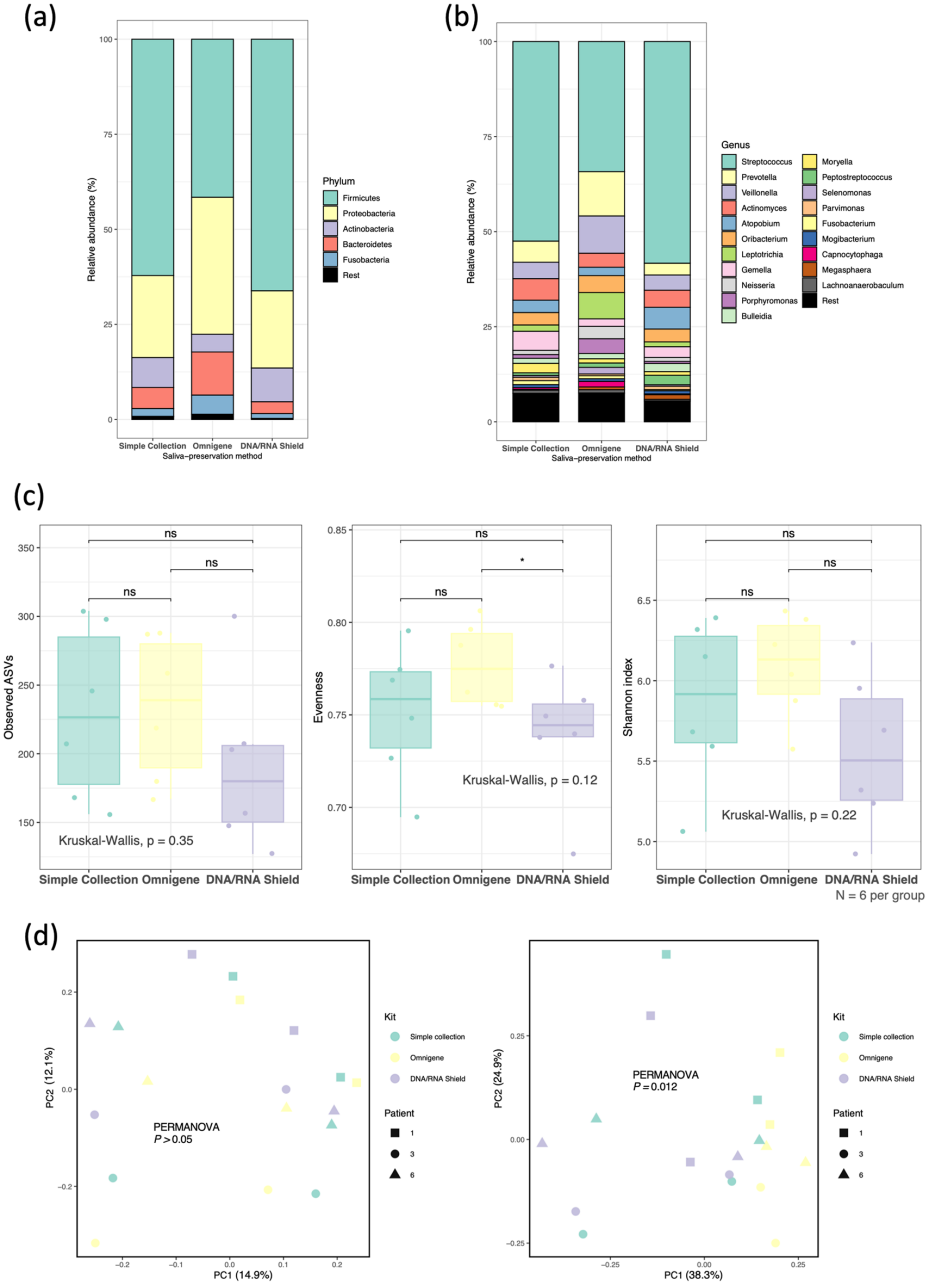
Comparison of the simple collection, OMNIgene, and DNA/RNA shield groups sequenced by short-read next-generation sequencing (SR-NGS). (**a, b**) Taxonomic barplots showing the taxa and their relative abundances. (**a**) Phylum level, (**b**) Genus level. The five most abundant phylum and 20 most abundant genera were shown based on the mean relative abundance of the three groups. (**c**) Box-and-whisker plots of the alpha diversity indices (observed features, Pielou’s evenness, and Shannon’s entropy). *ns* not significant; *: 0.01 ≤ *P* < 0.05 (**d**) Beta diversities (left: Jaccard distances, right: Bray–Curtis distances) as revealed by principal coordinate analysis (PCoA) plots.

To determine whether the type of salivary preservation method affected the taxonomic compositions, we estimated the relative taxon abundances at the phylum and genus levels. At the phylum level, Firmicutes and Proteobacteria were the first and second most abundant taxa in all three groups. At the genus level, *Streptococcus* was the most common taxon in all groups. *Atopobium* showed the highest abundance in the DNA/RNA shield group; *Prevotella*, *Veillonella*, *Leptotrichia*, and *Porphyromonas* were the most abundant in the OMNIgene group, while *Gemella* was in the simple collection group (Fig. 3a, b, and S3).

In terms of microbiome richness and evenness, no difference in the observed ASVs or Shannon’s entropy of any group was apparent. The group differences in Pielou’s evenness were also insignificant. However, the OMNIgene group showed more evenness than did DNA/RNA shield group (*P* = 0.037) (Fig. 3c). Beta diversity varied according to the Jaccard and Bray–Curtis distances. The Adonis test revealed no group difference in the Jaccard distances, while the OMNIgene group was well-differentiated from the other groups in the Bray–Curtis distances (*P* = 0.012) (Fig. 3d).

#### LR-NGS group

**Figure 4:**
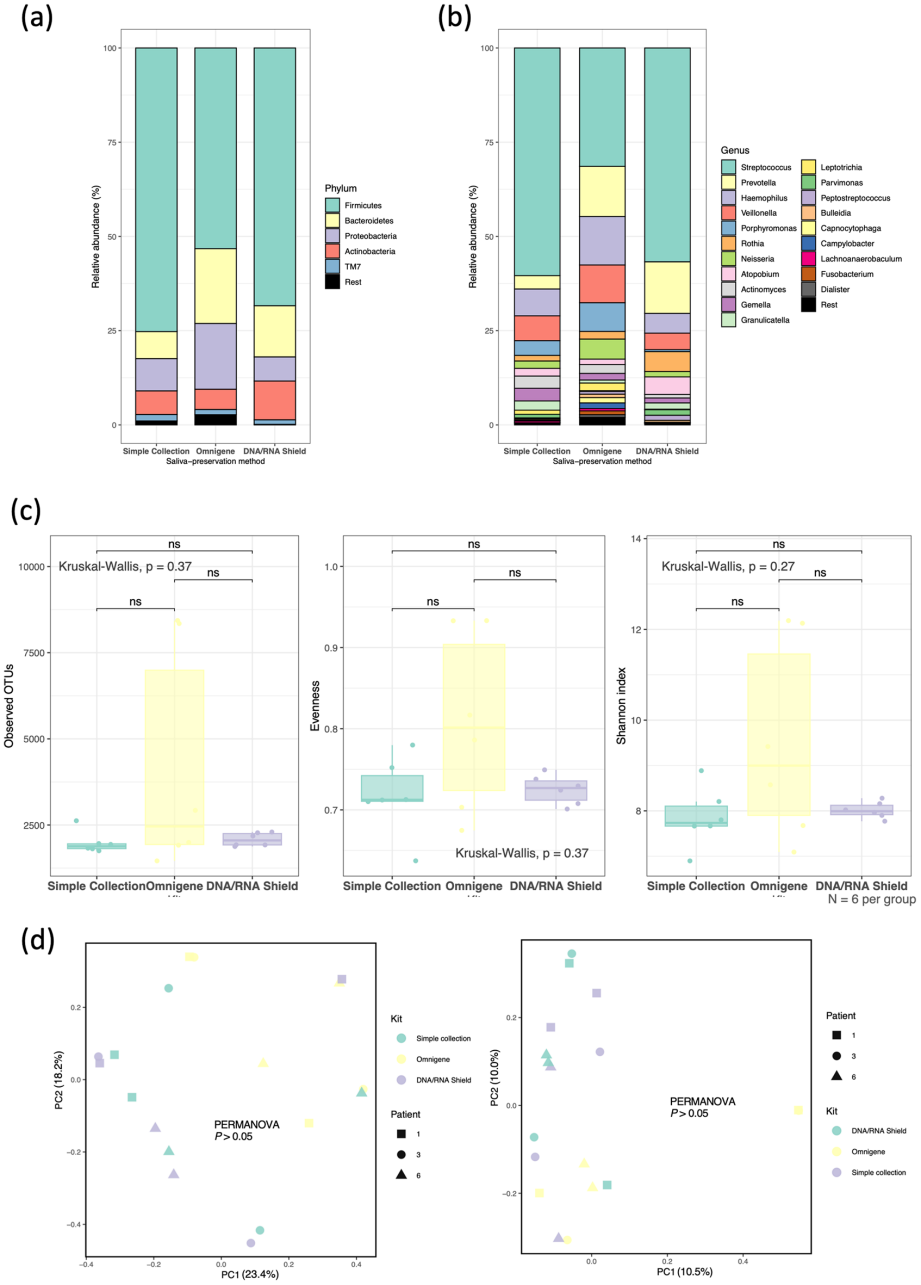
Comparison of the simple collection, OMNIgene, and DNA/RNA shield groups sequenced by long-read next-generation sequencing (LR-NGS). (**a, b**) Taxonomic barplots showing the taxa and their relative abundances. (**a**) Phylum level, (**b**) Genus level. The five most abundant phylum and 20 most abundant genera were shown based on the mean relative abundance of the three groups. (**c**) Box-and-whisker plots of the alpha diversity indices (observed features, Pielou’s evenness, and Shannon’s entropy). *ns* not significant (**d**) Beta diversities (left: Jaccard distances, right: Bray–Curtis distances) as revealed by principal coordinate analysis (PCoA) plots.

The relative taxon abundances of each saliva-preservation method are shown in Fig. 4a, b. At the phylum level, Firmicutes and Bacteroidetes were the first and second most abundant taxa in all three groups. At the genus level, *Streptococcus* was the most common taxon in all groups. *Porphyromonas, Haemophilus, Porphyromonas Gemella, Actinomyces*, and *Neisseria* were lacking in samples aliquoted into the DNA/RNA shield; *Prevotella* and *Rothia* were least abundant in the simple collection group, while *Atopobium* was in the OMNIgene group.

The alpha and beta diversity analyses revealed that the OMNIgene, DNA/RNA shield, and simple collection groups had similar species richness and evenness levels; the differences in taxonomic composition were insignificant (Fig. 4c, d).

## Discussion

Data comparability is essential when evaluating different studies or the long-term results of single studies, since noncomparability leads to inconsistent findings and erroneous conclusions. Whereas standardization can be achieved by ensuring traceability to the International System of Units, harmonization is achieved by ensuring traceability to an agreed reference system with the verification of measurement comparability, which is dependent on analytical techniques^12^. And this is the reason why methods measuring creatinine or thyroid function have been refined and standardized^13,14^.

However, achieving data comparability is very difficult when evaluating microbiomes. The sample collection method used, preservation and storage, DNA extraction and sequencing, and data analysis may create bias^15^. This is why apparently similar studies come to different conclusions, and the reason microbiome studies show high-level methodological bias. Fortunately, the need for comparability of microbiome (especially gut microbiome) data is being recognized. Several protocols aim to control for variations in animal gut microbiota and standardize the conduct of clinical trials of dietary interventions, cardiometabolic diseases, and the gut microbiota^16,17^. The attempts for achieving comparability of oral microbiome are also being made, as mentioned in Introduction section.

In this study, we evaluated parameters that might influence oral microbiome data and compared the data of oral microbiome gathered by SR-NGS and LR-NGS, which is original to the best of our knowledge. We first compared drooling and mouthwash groups; in contrast to previous studies showing similar within-sample diversity between drooling and mouthwash samples, the alpha diversity indices (observed ASVs and Shannon’s entropy) were significantly higher in the latter group, regardless of the NGS method^10,18,19^. As the number of features reflects richness, and the Shannon’s entropy both richness and evenness, the richness of the mouthwash group was higher than that of the drooling group. The beta diversity results differed between the SR-NGS and LR-NGS groups; the compositions differed significantly when SR-NGS was used, whereas the compositions in the LR-NGS group did not. The difference between microbial compositions of drooling and mouthwash samples in the SR-NGS group was explained by the relatively rare genera, including *Actinobacillus, Bordetella, Corynebacterium, Cupriavidus, Lactobacillus, Methylobacterium, Olsenella, Pseudomonas,* and *Rothia* (Table 1 and Fig. 1b). The abundant genera, such as *Streptococcus*, *Prevotella*, and *Veillonella* showed no difference between the two groups; this result was consistent with the previous study conducted by Fan et al.^10^.

Higher richness may not always be desirable; the mouthwash flora may contain non-specific normal flora. Nevertheless, higher mouthwash richness has the potential of clinical utility. Mouthwash has been avoided by some in the belief that it dilutes the microbiome. However, the higher richness of mouthwash taxa obviates this concern. The higher richness can be interpreted as the possibility that mouthwash may more accurately reflect the oral microbiome. Mouthwash covers more parts of the oral cavity, while saliva from the oral cavity floor is only gathered in drooling method. Another possible factor is the influence of the Benzonase digestion step, which is used for host DNA depletion in microbiome DNA extraction methods, but also removes cell-free microbial DNA and leaves only nucleic acids inside intact microbial membranes; Previous studies that did not include a host DNA depletion step during DNA extraction showed little difference between the two collection methods^10, 18, 19^. Mouthwash is very useful in patients who do not create drool easily, such as those with xerostomia, autoimmune diseases (e.g., Sjogren’s syndrome), and systemic diseases (e.g., Parkinsonism and stroke). Considering these perspectives, we suggest that mouthwash is not inferior to drooling, especially for patients with drooling difficulties.

The alpha and beta diversities provided by the three different saliva-preservation methods used in this study were very similar. This suggests that simple collection could be used as the saliva-preservation method, which could be a great help to clinical laboratories hesitating to use expensive preservative-containing kits^20^. Still, the value of OMNIgene and DNA/RNA shield kits should not be undermined, since they are efficient when the interval between extraction and sequencing is long.

Before comparing the SR-NGS and LR-NGS data, we must address their innate differences. In the SR-NGS group, the maximum sequence length of iSeq was 2 × 150 bp (paired ends), and the V4 hypervariability regions of 16S rRNA were amplified and sequenced. The ASV feature table was produced by denoising (removing erroneous sequences followed by counting the variant amplicon sequences). In the LR-NGS group, the entire 16S rRNA regions were amplified and sequenced. As the error rate of long-read sequencing is higher than that of short-read sequencing, QIIME2 was unable to create an ASV feature table, instead generating only a conventional OTU table.

SR-NGS and LR-NGS revealed similar results when analyzing taxa. At the phylum level, Firmicutes, Proteobacteria, Actinobacteria, and Bacteroidetes were dominant in two groups. However, the relative abundances differed; Proteobacteria and Bacteroidetes were the second most abundant taxa in the SR-NGS and LR-NGS groups, respectively. Similarities of taxon types and differences in relative abundances were also apparent at the genus level. In terms of diversity, the alpha diversity indices were higher in the mouthwash than drooling in both SR-NGS and LR-NGS groups. When the three different saliva-preservation methods were tested, both SR-NGS and LR-NGS did not exhibit any significant difference. However, both beta diversity indices were significantly higher in the mouthwash group sequenced by SR-NGS, while no difference was shown in LR-NGS group.

We suggest that the difference is attributable to the 16S rRNA regions sequenced, as the taxonomic outcomes can be differed by the region analyzed. Katiraei et al.^21^ reporting the differences in relative bacterial abundances, alpha, and beta diversity between 16s rRNA V4 region data and full-length data supports our results. Also, as the target sites of SR-NGS and LR-NGS differed, so too did the primers, which might influenced the sequencing outcomes by not covering V4 flanking region of all bacteria^21^. However, this seemed to be unimportant given that the taxa of both groups were similar^21^.

Some limitations of our study should be pointed out. The differences between the ASV and OTU feature tables might partly explain the diversity differences. ASV methods are more sensitive and specific than OTU methods, and are better able to reveal ecological patterns^22^. It is possible that the OTU table for the LR-NGS group contained erroneous sequences, thereby skewing the phylogenic construction and diversity analysis. Moreover, our study design lacks negative control. However, for NGS-based microbiome studies, it is impossible to differentiate between actual contamination and index hopping, a phenomenon in that index sequences assigned to a specific sample are incorrectly assigned to the other samples in a multiplexed pool, which can make the controls potentially useless. This could be avoided by sequencing negative controls in separate runs, but it is unlikely to be feasible as it increases the sequencing cost to an unacceptable level. To achieve standardization of the method, further studies including larger sample sizes should be conducted, along with other conditions associated with saliva sampling, such as unstimulated versus stimulated saliva^23^.

In conclusion, we aimed to optimize salivary sampling prior to oral microbiome analyses. Mouthwash samples and a simple sample collection method were not inferior to drooling and the use of preservatives, respectively. The convenience and cost-effectiveness of mouthwash and simple collection would be helpful to clinical operators and lab workers. Results vary depending on the type of NGS, and the strengths and weaknesses of SR-NGS and LR-NGS require further analysis using more samples to find the optimal usage of each NGS techniques in microbiome analysis. Similarly, larger studies are required to confirm the non-inferiority of mouthwash and simple collection methods, along with the studies comparing the data of healthy one’s microbiome and the patient’s using mouthwash and simple collection.

## Methods

### Sample collection

**Figure 5:**
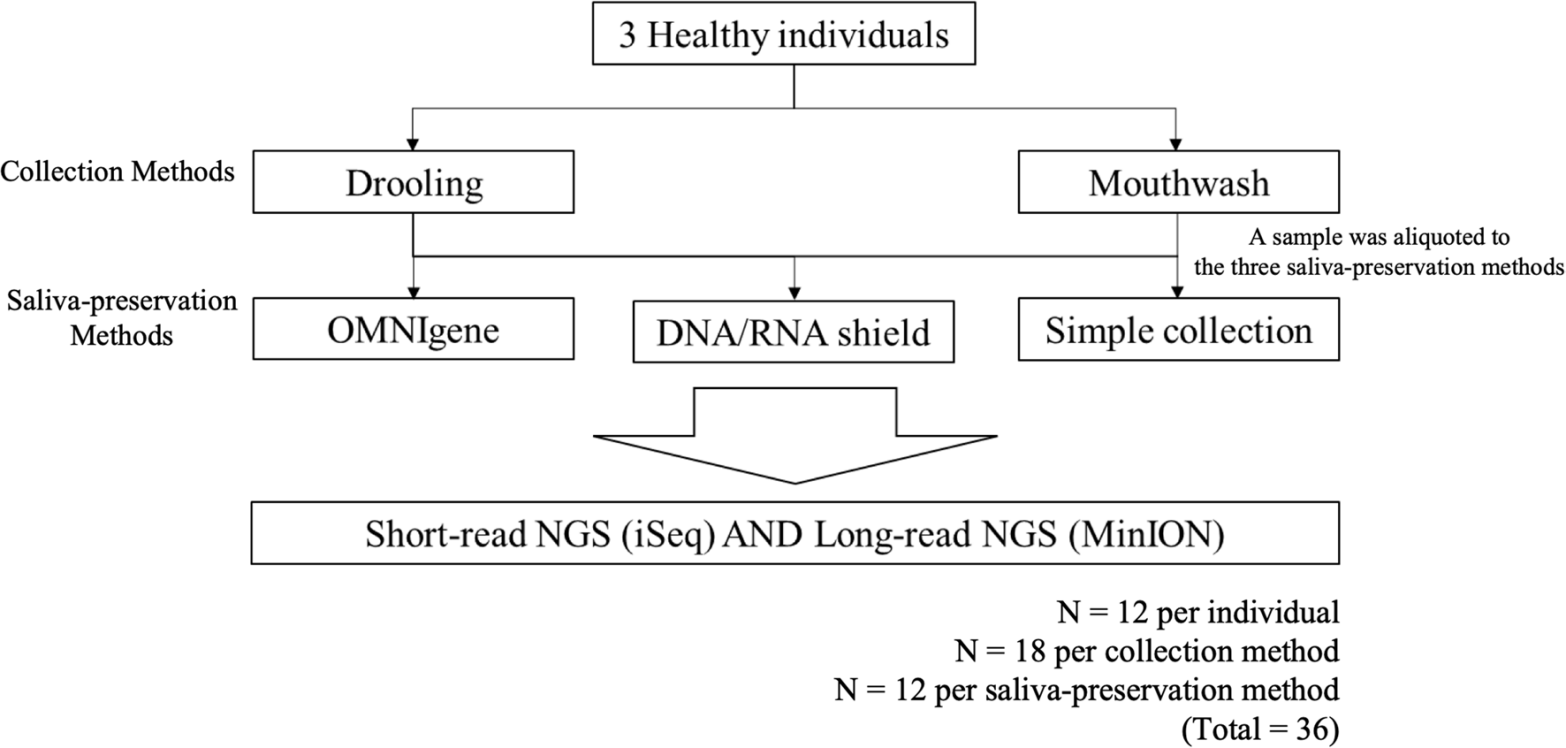
A simplified flow chart of the sample collection process. Three healthy individuals were enrolled; saliva was collected as drooling or mouthwash. The samples were divided into three groups depending on the type of saliva-preservation methods (OMNIgene, DNA/RNA shield, or simple collection). Twenty samples (18 from healthy individuals and 2 mock samples) were collected and sequenced. *NGS* next-generation sequencing.

Drooling and mouthwash samples were collected from three healthy individuals who provided written informed consent. Drooling sample (10–15 mL) was collected by asking the volunteers to accumulate saliva on the floor of the mouth and then expectorate it^24^. One hour elapsed between drooling and mouthwash to allow the oral microbiome to be restored. 10 mL of mouthwash samples were collected by asking the volunteers to rinse the oral cavity with 0.9% normal saline for about 30 s, allowing them to swash, and expectorate it into a 10 mL conical tube. The samples were aliquoted to the three saliva-preservation methods right after the sample was collected: 1 mL into the OMNIgene tube (DNA Genotek Incorporation, Ontario, Canada), 2 mL into the DNA/RNA shield tube (Zymo Research, California, USA), and 1 mL into the simple collection tube without preservatives. The former two tubes contain stabilization buffers allowing immediate ambient temperature homogenization and stabilization. A total of 18 samples from the three individuals were collected by the two sample collection methods (drooling and mouthwash) using the three saliva-preservation methods. Two samples from a mock group (for which sequencing data were available) served as controls. A schematic of the sample collection process is shown in Fig. 5. The study adhered to the guidelines of the Declaration of Helsinki and was reviewed by the Institutional Review Board (IRB) of Seoul National University Bundang Hospital (IRB no. B-2107-695-301).

### DNA extraction and 16s rRNA gene sequencing

#### SR-NGS using an Illumina platform

The samples were stored at − 80 °C until the DNA extraction. 1 mL of the thawed whole sample was subjected to DNA extraction using QIAamp DNA microbiome kits (QIAGEN, Venlo, the Netherlands) following the manufacturer’s protocol. For amplicon PCR targeting the V4 hypervariable region of 16S rRNA genes, KAPA HiFi HotStart ReadyMix PCR kits (Roche, Basel, Switzerland) were used following the manufacturers’ instructions. Libraries were constructed with NextEra XT DNA library preparation kits (Illumina Inc., San Diego, CA, USA) and pooled to final loading concentrations of 50 pM. Sequencing was performed on the Illumina iSeq 100 platform using 100 i1 REAGENT cartridges (2 × 150 bp paired-ends); the control library was that of the PhiX Control kit ver. 3 (Illumina).

#### LR-NGS using the nanopore platform

For amplicon PCR targeting the entire 16S rRNA coding regions, KAPA HiFi HotStart ReadyMix PCR kits were used. For DNA repair and end-preparation, NEBNext FFPE DNA Repair Mix (New England Biolabs, Ipswich, MA, USA) and the NEBNext Ultra II End Repair/dA-Tailing Module (New England Biolabs) were used. Barcodes and sequencing adapters were ligated using Native Barcoding Expansion 1–12 kits (EXP-NBD104; Oxford Nanopore Technologies, Oxford, UK) and ligation sequencing kits (SQK-LSK109; Oxford Nanopore Technologies) following the manufacturer’s protocols. Libraries were sequenced using the R9.4.1 flow cell (FLO-MIN106D), MinION software (Oxford Nanopore Technologies) and a flow cell priming kit (EXP-FLP002; Oxford Nanopore Technologies).

### Bioinformatic and statistical analysis

#### SR-NGS

FastQC software (Babraham Bioinformatics, Babraham, UK) was used to assess the quality of demultiplexed fastq data. After adapters were trimmed, a Divisive Amplicon Denoising Algorithm (DADA) 2-based pipeline^25^ was applied using QIIME2 21.8^26^. Feature ASV tables were produced by quality-based filtering and trimming, read dereplication and ASV inference, paired-end merging, and removal of chimeric ASVs. The ASV tables were normalized by rarefaction, to correct artifactual biases prior to downstream analyses. To estimate microbiome taxonomic compositions, the sequences were taxonomically classified against the Greengenes 13_8 99% list^27^ using scikit-learn (i.e., the pre-trained naive Bayes machine-learning classifier of the q2-feature-classifier plugin). The relative abundances of microbiome phyla, genera, and species were plotted using R software (ver. 4.1.2; R Development Core Team, Vienna, Austria); all data visualizations and statistical analyses were also done using R software.

Alpha diversity indices (observed features, Shannon’s entropy, Pielou’s evenness, and the Faith’s phylogenetic diversity) were calculated to evaluate microbiome richness and evenness among groups^28^. We used the Wilcoxon rank-sum test and Kruskal–Wallis H-test (with Benjamini–Hochberg FDR correction) to determine whether alpha diversity differed significantly among the groups. Beta diversity indices (the Jaccard and Bray–Curtis distances, and unweighted and weighted UniFrac distance matrices) were obtained and visualized using PCoA^29^. We performed permutation multivariate analysis of variance (PERMANOVA) using Adonis test to compare microbiome composition between groups^30^. Analysis of Composition of Microbiomes with Bias Correction (ANCOM-BC), which estimates the unknown sampling fractions and corrects the bias induced by their differences through a log-linear regression model was used for differential abundance analysis^31^. The correction for multiple testing was performed using the Holm-Bonferroni method and statistical significance was thus determined as an adjusted *p* value < 0.05.

#### LR-NGS

For base-calling and demultiplexing of long reads, Guppy Basecaller (ver. 4.2.2 (Oxford Nanopore Technologies) was used^32^. After data quality was evaluated using EPI2ME software, Trimmomatic ver. 0.40 was used to trim the adapters and truncate reads to 1400 bp^33^. During QIIME2-based analysis of full-length 16S rRNA sequences generated by LR-NGS, OTU clustering at 85% identity was performed, rather than ASV-based denoising, using the q2ONT pipeline (https://github.com/DeniRibicic/q2ONT). After dereplicating trimmed reads and filtering away chimeric sequences, open-reference clustering (85% identity) was conducted using the VSEARCH algorithms^34^. Downstream analyses (taxonomic classification, diversity, and differential abundance analyses) were performed as described above for the SR-NGS data.

## Supplementary Information

Supplementary Information.

## Data Availability

The sequencing data of this study can be accessed at the Sequence Read Archives (SRA) (Accession no. PRJNA889989). Additional data and materials are available upon reasonable request from the corresponding author.
