# Supplementary material for: Factors influencing oral microbiome analysis: from saliva sampling methods to next-generation sequencing platforms
Source: Sci Rep. 2023 Jun 21;13:10086. doi: 10.1038/s41598-023-37246-2 (PMC10284873; doi:10.1038/s41598-023-37246-2)
Supplement: Supplementary file 1 — Supplementary Information. [file 41598_2023_37246_MOESM1_ESM.docx]

**Supplementary Information**

**Supplementary Figure S1**. Taxonomic barplots showing the taxa and their relative abundances in each sample of the drooling and mouthwash groups sequenced by short-read next-generation sequencing (SR-NGS). Upper; Phylum level, lower; Genus level. The five most abundant phylum and 20 most abundant genera were shown based on the mean relative abundance of the two groups.

**Supplementary Figure S2**. Taxonomic barplots showing the taxa and their relative abundances in each sample of the drooling and mouthwash groups sequenced by long-read next-generation sequencing (LR-NGS). Upper; Phylum level, lower; Genus level. The five most abundant phylum and 20 most abundant genera were shown based on the mean relative abundance of the two groups.

**Supplementary Figure S3**. Taxonomic barplots showing the taxa and their relative abundances in each sample of the simple collection, OMNIgene, and DNA/RNA shield groups sequenced by short-read next-generation sequencing (SR-NGS). Upper; Phylum level, lower; Genus level. The five most abundant phylum and 20 most abundant genera were shown based on the mean relative abundance of the three groups.

**Supplementary Figure S4**. Taxonomic barplots showing the taxa and their relative abundances in each sample of the simple collection, OMNIgene, and DNA/RNA shield groups sequenced by long-read next-generation sequencing (LR-NGS). Upper; Phylum level, lower; Genus level. The five most abundant phylum and 20 most abundant genera were shown based on the mean relative abundance of the three groups.

subject-01

subject-02

subject-03

subject-01

subject-02

subject-03

subject-01

subject-02

subject-03
